# Supplementary material for: Extraction and Purification of Highly Active Astaxanthin from Corynebacterium glutamicum Fermentation Broth
Source: Mar Drugs. 2023 Oct 11;21(10):530. doi: 10.3390/md21100530 (PMC10608131; doi:10.3390/md21100530)
Supplement: Supplementary file 1 [file marinedrugs-21-00530-s001.zip › marinedrugs-2654391-supplementary.pdf]

Supplementary materials

Extraction and purification of highly active astaxanthin from *Corynebacterium glutamicum* fermentation broth

Jan Seeger, Volker F. Wendisch and Nadja A. Henke *

Genetics of Prokaryotes, CeBiTec, Bielefeld University, Germany
* Correspondence: n.henke@uni-bielefeld.de

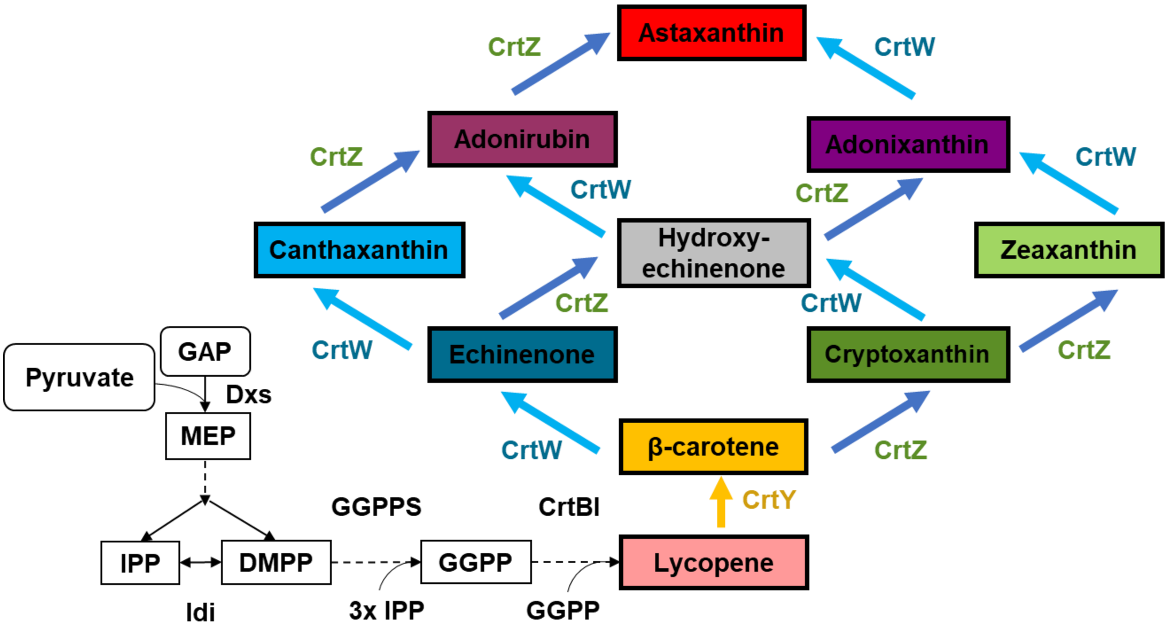

Figure S1. Astaxanthin biosynthesis pathway in *C. glutamicum*. Scheme of the astaxanthin biosynthesis pathway based on the central carbon metabolism, including respective precursor carotenoids. Heterologous expression of CrtY, CrtW, CrtZ. Enzymes are given in abbreviated form next to the reaction. GAP: Glyceraldehyde 3-phosphate; DXS: 1-deoxy-D-xylulose-5-phosphate synthase; MEP: 2-C-methyl-D-erythritol 4-phosphate pathway; IPP: Isopentenyl diphosphate; DMAPP: Dimethylallyl pyrophosphate; Idi: Isopentenyl pyrophosphate isomerase; GGPPS: Geranylgeranyl pyrophosphate synthase; GGPP: Geranylgeranyl pyrophosphate; CrtB: Phytoene synthase; CrtI: Phytoene desaturase; CrtY: Lycopene cyclase; CrtW: β-carotene ketolase; CrtZ: β-carotene hydroxylase.

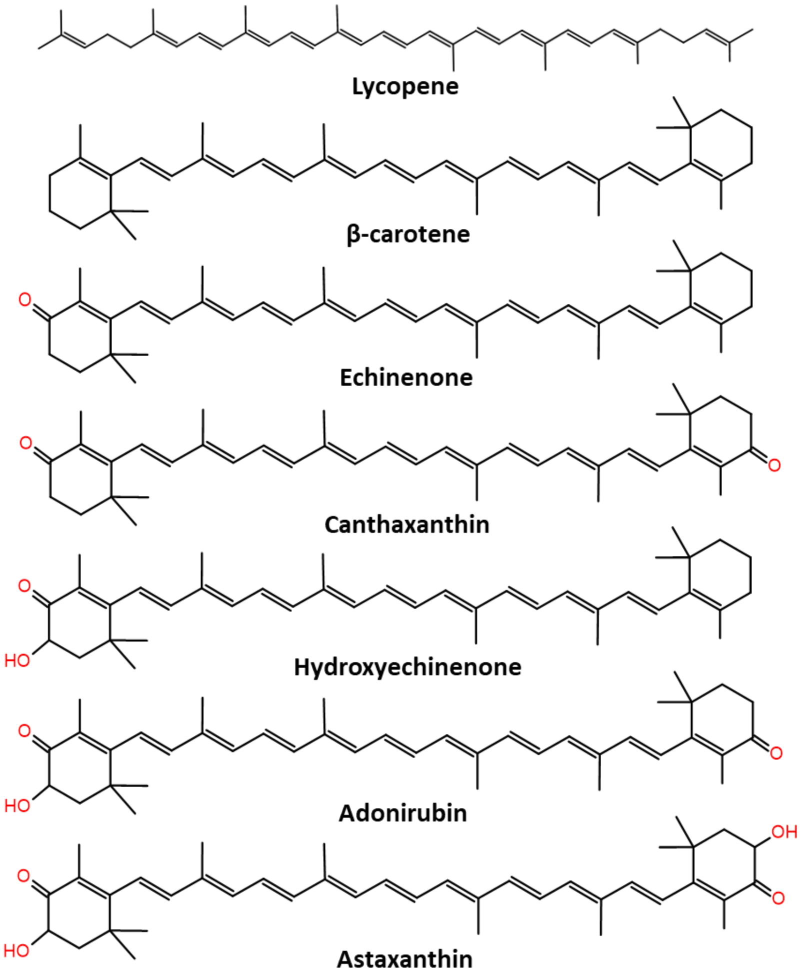

Figure S2. Structure of carotenoids detected by HPLC in this study. Oxy-functionalization is highlighted in red.
